# Bis{μ-2-[(dimethyl­amino)­meth­yl]benzene­seleno­lato}bis­[chloridopalladium(II)] dichloromethane hemisolvate

**DOI:** 10.1107/S1600536811055322

**Published:** 2012-01-07

**Authors:** Tapash Chakravorty, Harkesh B. Singh, Ray J. Butcher

**Affiliations:** a Department of Chemistry, Indian Institute of Technology Bombay, Powai, Mumbai 400 076, India; b Department of Chemistry, Howard University, 525 College Street NW, Washington, DC 20059, USA

## Abstract

The asymmetric unit of the title compound, [Pd_2_(C_9_H_12_NSe)_2_Cl_2_]·0.5CH_2_Cl_2_, contains two half-mol­ecules, each lying on a twofold axis; each mol­ecule is chiral and of the same enanti­omer. This is only possible as the mol­ecule has a hinged *cis* arrangement about the Pd^2+^ coordination spheres. For this hinged dimeric structure, the angles between the two coordination planes in each mol­ecule are 15.02 (5) and 14.91 (5)°. This hinged *cis* arragement also allows the two mol­ecules to form pairs linked by secondary inter­actions between the Pd and Se atoms [3.4307 (9) and 3.4317 (9) Å] of adjoining mol­ecules, leading to an overall tetra­meric structure. During the refinement stages, it was noticed that there were dichloromethane solvent mol­ecules present disordered about a twofold axis. After unsuccessful attempts were made to model this, they were removed using SQUEEZE.

## Related literature

For applications of organoselenide and organotelluride ligands in materials science, see: Morley *et al.* (2006 ▶); Ford *et al.* (2004 ▶). For structures of dimeric Se-bridged Pd derivatives, see: Nakata *et al.* (2009 ▶); Chakraborty *et al.* (2011 ▶); Oilunkaniemi *et al.* (1999 ▶, 2001 ▶); Brown & Corrigan (2004 ▶); Dey *et al.* (2006 ▶) and for structures of dimeric Te-bridged Pd derivatives, see: Oilunkaniemi *et al.* (2000 ▶); Kaur *et al.* (2009 ▶); Dey *et al.* (2006 ▶). For the use of the SQUEEZE routine in *PLATON*, see: Spek (2009 ▶).
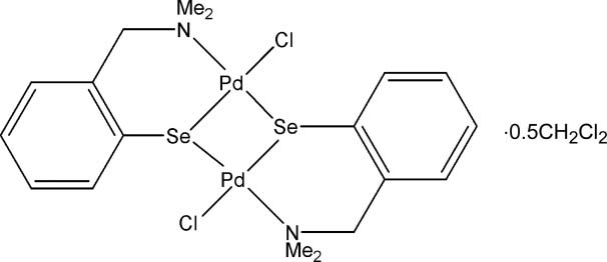


## Experimental

### 

#### Crystal data

[Pd_2_(C_9_H_12_NSe)_2_Cl_2_]·0.5CH_2_Cl_2_
*M* *_r_* = 752.47
Orthorhombic, 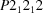

*a* = 14.2119 (1) Å
*b* = 14.7895 (1) Å
*c* = 12.0968 (1) Å
*V* = 2542.59 (3) Å^3^
*Z* = 4
Mo *K*α radiation
μ = 4.60 mm^−1^
*T* = 293 K
0.35 × 0.24 × 0.12 mm

#### Data collection

Oxford Diffraction Xcalibur Ruby Gemini diffractometer
Absorption correction: multi-scan (*CrysAlis PRO*; Oxford Diffraction, 2007 ▶) *T*_min_ = 0.655, *T*_max_ = 1.000
22757 measured reflections
5323 independent reflections
4971 reflections with *I* > 2σ(*I*)
*R*_int_ = 0.073

#### Refinement

*R*[*F* ^2^ > 2σ(*F* ^2^)] = 0.051
*wR*(*F* ^2^) = 0.131
*S* = 1.06
5323 reflections
240 parameters
H-atom parameters constrained
Δρ_max_ = 1.29 e Å^−3^
Δρ_min_ = −1.45 e Å^−3^
Absolute structure: Flack (1983 ▶), 2261 Friedel pairs
Flack parameter: 0.015 (13)

### 

Data collection: *CrysAlis PRO* (Oxford Diffraction, 2007 ▶); cell refinement: *CrysAlis PRO*; data reduction: *CrysAlis PRO*; program(s) used to solve structure: *SHELXS97* (Sheldrick, 2008 ▶); program(s) used to refine structure: *SHELXL97* (Sheldrick, 2008 ▶); molecular graphics: *SHELXTL* (Sheldrick, 2008 ▶); software used to prepare material for publication: *SHELXTL*.

## Supplementary Material

Crystal structure: contains datablock(s) I, global. DOI: 10.1107/S1600536811055322/hg5157sup1.cif

Structure factors: contains datablock(s) I. DOI: 10.1107/S1600536811055322/hg5157Isup2.hkl

Additional supplementary materials:  crystallographic information; 3D view; checkCIF report

## Figures and Tables

**Table 1:** Hydrogen-bond geometry (Å, °)

*D*—H⋯*A*	*D*—H	H⋯*A*	*D*⋯*A*	*D*—H⋯*A*
C5*A*—H5*AA*⋯Cl1*A*^i^	0.93	2.91	3.782 (10)	156
C7*A*—H7*AA*⋯Cl1*A*^i^	0.97	2.94	3.853 (8)	158
C9*A*—H9*AC*⋯Cl1*A*	0.96	2.79	3.325 (10)	116
C5*B*—H5*BA*⋯Cl1*B*^ii^	0.93	2.94	3.808 (11)	156
C8*B*—H8*BB*⋯Cl1*B*	0.96	2.80	3.347 (11)	117

Symmetry codes: (i) 
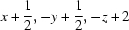
; (ii) 
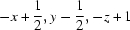
.

**Fig. 1.**
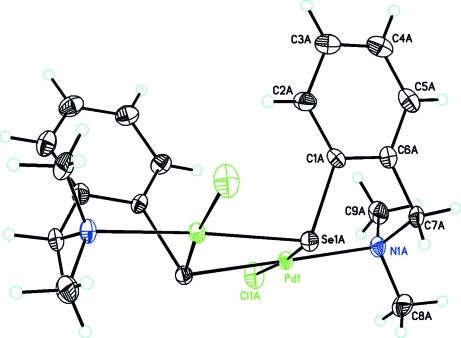
The structure of one of the two molecules of the asymmetric unit showing the hinged cis arrangement of the two Pd coordination planes. The two halves of the molecule are related by 1 - x, 1 - y, z.

**Fig. 2.**
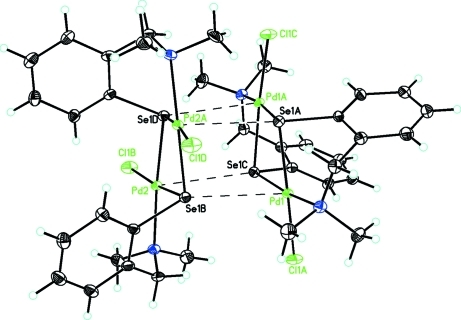
The association of two dimeric units into a tetramer via matching and complementary secondary interactions between the Pd and Se of adjoining units. These interactions are shown by dashed lines.

**Fig. 3.**
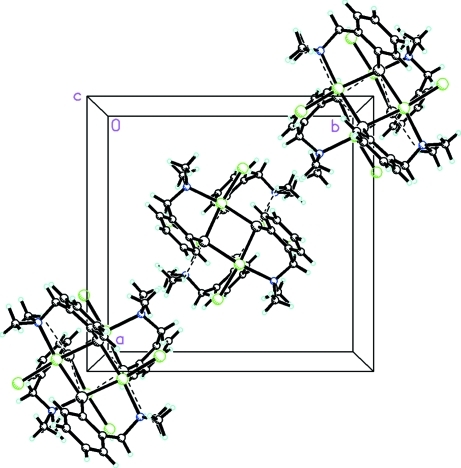
Showing the packing of the tetrameric units. Secondary interactions between Pd and Se shown by dashed lines.

## supplementary crystallographic information

### Comment

The coordination chemistry of transition metal complexes with both organoselenide and organotelluride ligands is a rapidly growing area due to the ability of the resulting complexes to find applications in materials science (Morley *et al.*, 2006; Ford *et al.*, 2004), and investigations of oxidation additive to low valent transition metal centers. In addition to this, organotellurium compounds have been used in catalytic carbon-carbon formation. Bridged dimers of palladium mediated by Se (Nakata *et al.*, 2009; Chakraborty *et al.*, 2011; Oilunkaniemi *et al.*, 1999; Oilunkaniemi *et al.*, 2001; Brown & Corrigan, 2004; Dey *et al.*, 2006) or Te (Oilunkaniemi *et al.*, 2000; Kaur *et al.*, 2009; Dey *et al.*, 2006) have been previously reported. Such dimers involving two square planar coordination spheres can adopt either a coplanar or hinged arrangement. The arrangement of the donor ligands with respect to the bridging plane can be *cis* or *trans*. In the case of a hinged *cis* arrangemnt the possibility of chirality exisits. While the majority of previously determined Se/Te bridged Pd dimeric structures are both coplanar and *trans*, there have been a small number which exhibit either a hinged or *cis* arrangment of ligands about the bridging plane (Kaur *et al.*, 2009; Oilunkaniemi *et al.*, 2000). However, in no previous case has this resulted in a chiral structure.

The title compound, bis[chlorido-(µ(Se)-2-dimethylaminomethylbenzeneselenolate)palladium(II)], C_18_H_24_Cl_2_N_2_Pd_2_Se_2_, crystallizes in the chiral orthorhombic space group, *P*2_1_2_1_2. The asymmetric unit contains 2 half molecules, each lying on a 2-fold axis and each molecule is chiral and of the same enantiomer. This is only possible as the molecule has a hinged *cis* arrangement about the Pd coordination spheres (Fig. 1). For this hinged dimeric structure the angles between the two coordination planes in each molecule are 15.02 (5) and 14.91 (5)° respectively. This hinged *cis* arragement also allows the two molecules to form pairs linked by secondary interactions between the Pd and Se of an adjoining molecule (Fig. 2) leading to a tetrameric overall structure. Apart from this the Pd—Se, Pd—Cl and Pd—N bond lengths are in the normal ranges.

### Experimental

The ligand and complex were prepared using previously reported methods (Chakraborty *et al.*, 2011). Crystallization of the selenolate was done at ambient temperature from dichloromethane/hexane (2:1).

### Refinement

H atoms were placed in geometrically idealized positions and constrained to ride on their parent atoms with C—H distances of 0.95 - 0.97 Å [*U*_iso_(H) = 1.2*U*_eq_(CH, CH_2_) [*U*_iso_(H) = 1.5*U*_eq_(CH_3_)]. During the refinement stages it was noticed that there were disordered solvent molecules present. The solvent molecule is CH_2_Cl_2_ and it is disordered about a 2-fold axis. After unsuccessful attempts were made to model this, it was removed using the SQUEEZE routine from *PLATON* (Spek, 2009).

### Crystal data

**Table:**

[Pd_2_(C_9_H_12_NSe)_2_Cl_2_]·0.5CH_2_Cl_2_	*F*(000) = 1444
*M**_r_* = 752.47	*D*_x_ = 1.966 Mg m^−^^3^
Orthorhombic, *P*2_1_2_1_2	Mo *K*α radiation, λ = 0.71073 Å
Hall symbol: P 2 2ab	Cell parameters from 16933 reflections
*a* = 14.2119 (1) Å	θ = 4.7–77.4°
*b* = 14.7895 (1) Å	µ = 4.60 mm^−^^1^
*c* = 12.0968 (1) Å	*T* = 293 K
*V* = 2542.59 (3) Å^3^	Prism, orange
*Z* = 4	0.35 × 0.24 × 0.12 mm

### Data collection

**Table:**

Oxford Diffraction Xcalibur Ruby Gemini diffractometer	5323 independent reflections
Radiation source: fine-focus sealed tube	4971 reflections with *I* > 2σ(*I*)
graphite	*R*_int_ = 0.073
Detector resolution: 10.5081 pixels mm^-1^	θ_max_ = 26.8°, θ_min_ = 2.6°
ω scans	*h* = −17→17
Absorption correction: multi-scan (CrysAlis PRO; Oxford Diffraction, 2007)	*k* = −18→18
*T*_min_ = 0.655, *T*_max_ = 1.000	*l* = −15→14
22757 measured reflections	

### Refinement

**Table:**

Refinement on *F*^2^	Hydrogen site location: inferred from neighbouring sites
Least-squares matrix: full	H-atom parameters constrained
*R*[*F*^2^ > 2σ(*F*^2^)] = 0.051	*w* = 1/[σ^2^(*F*_o_^2^) + (0.0803*P*)^2^ + 6.8337*P*] where *P* = (*F*_o_^2^ + 2*F*_c_^2^)/3
*wR*(*F*^2^) = 0.131	(Δ/σ)_max_ = 0.001
*S* = 1.06	Δρ_max_ = 1.29 e Å^−^^3^
5323 reflections	Δρ_min_ = −1.45 e Å^−^^3^
240 parameters	Extinction correction: *SHELXL97* (Sheldrick, 2008), Fc^*^=kFc[1+0.001xFc^2^λ^3^/sin(2θ)]^-1/4^
0 restraints	Extinction coefficient: 0.0067 (8)
Primary atom site location: structure-invariant direct methods	Absolute structure: Flack (1983), 2261 Friedel pairs
Secondary atom site location: difference Fourier map	Flack parameter: 0.015 (13)

### Special details

**Table:**

Experimental. The structure of the Te analog was also determined, but at low temperature. This compound is isostructural and isomorphous with the Se compound but in this case the solvent was ordered. An Acta E submission for this structure has been made and it is currently under review (jj2116).
Geometry. All e.s.d.'s (except the e.s.d. in the dihedral angle between two l.s. planes) are estimated using the full covariance matrix. The cell e.s.d.'s are taken into account individually in the estimation of e.s.d.'s in distances, angles and torsion angles; correlations between e.s.d.'s in cell parameters are only used when they are defined by crystal symmetry. An approximate (isotropic) treatment of cell e.s.d.'s is used for estimating e.s.d.'s involving l.s. planes.
Refinement. Refinement of *F*^2^ against ALL reflections. The weighted *R*-factor *wR* and goodness of fit *S* are based on *F*^2^, conventional *R*-factors *R* are based on *F*, with *F* set to zero for negative *F*^2^. The threshold expression of *F*^2^ > σ(*F*^2^) is used only for calculating *R*-factors(gt) *etc*. and is not relevant to the choice of reflections for refinement. *R*-factors based on *F*^2^ are statistically about twice as large as those based on *F*, and *R*- factors based on ALL data will be even larger.

### Fractional atomic coordinates and isotropic or equivalent isotropic displacement parameters (Å^2^)

**Table:**

	*x*	*y*	*z*	*U*_iso_*/*U*_eq_	
Pd1	0.53556 (3)	0.38325 (3)	1.09852 (5)	0.03277 (16)	
Pd2	0.37837 (4)	0.46542 (3)	0.40196 (5)	0.03435 (17)	
Se1A	0.60568 (5)	0.52886 (5)	1.11898 (6)	0.0382 (2)	
Se1B	0.53041 (5)	0.39888 (5)	0.38141 (6)	0.0396 (2)	
Cl1A	0.44999 (17)	0.24995 (14)	1.0741 (3)	0.0633 (7)	
Cl1B	0.23941 (16)	0.54777 (17)	0.4277 (3)	0.0681 (7)	
N1A	0.6735 (4)	0.3189 (4)	1.0840 (6)	0.0423 (15)	
N1B	0.3119 (5)	0.3330 (4)	0.4160 (6)	0.0450 (15)	
C1A	0.6601 (5)	0.5140 (5)	0.9762 (7)	0.0373 (15)	
C2A	0.6352 (6)	0.5673 (6)	0.8858 (7)	0.0481 (18)	
H2AA	0.5904	0.6126	0.8934	0.058*	
C3A	0.6788 (7)	0.5518 (7)	0.7820 (8)	0.056 (2)	
H3AA	0.6617	0.5858	0.7205	0.067*	
C4A	0.7474 (8)	0.4854 (7)	0.7735 (8)	0.062 (3)	
H4AA	0.7769	0.4760	0.7058	0.074*	
C5A	0.7725 (7)	0.4333 (7)	0.8620 (9)	0.058 (2)	
H5AA	0.8198	0.3903	0.8540	0.070*	
C6A	0.7280 (6)	0.4437 (6)	0.9648 (7)	0.0446 (18)	
C7A	0.7490 (5)	0.3843 (6)	1.0628 (7)	0.0443 (17)	
H7AA	0.8073	0.3520	1.0496	0.053*	
H7AB	0.7576	0.4219	1.1277	0.053*	
C8A	0.6919 (8)	0.2716 (7)	1.1902 (9)	0.064 (3)	
H8AA	0.7536	0.2452	1.1884	0.096*	
H8AB	0.6458	0.2249	1.2007	0.096*	
H8AC	0.6882	0.3141	1.2500	0.096*	
C9A	0.6742 (7)	0.2505 (7)	0.9954 (9)	0.060 (2)	
H9AA	0.7369	0.2278	0.9859	0.090*	
H9AB	0.6531	0.2775	0.9276	0.090*	
H9AC	0.6329	0.2016	1.0149	0.090*	
C1B	0.5136 (6)	0.3459 (5)	0.5257 (7)	0.0397 (16)	
C2B	0.5680 (6)	0.3721 (6)	0.6158 (7)	0.0479 (18)	
H2BA	0.6138	0.4166	0.6083	0.057*	
C3B	0.5523 (7)	0.3301 (7)	0.7180 (8)	0.058 (2)	
H3BA	0.5878	0.3469	0.7792	0.070*	
C4B	0.4861 (8)	0.2656 (7)	0.7282 (9)	0.065 (3)	
H4BA	0.4768	0.2382	0.7965	0.078*	
C5B	0.4311 (7)	0.2390 (6)	0.6379 (8)	0.055 (2)	
H5BA	0.3868	0.1932	0.6462	0.066*	
C6B	0.4427 (7)	0.2813 (5)	0.5347 (8)	0.0466 (19)	
C7B	0.3803 (7)	0.2593 (5)	0.4390 (8)	0.0490 (19)	
H7BA	0.4187	0.2492	0.3738	0.059*	
H7BB	0.3463	0.2039	0.4545	0.059*	
C8B	0.2391 (7)	0.3300 (8)	0.5030 (9)	0.061 (3)	
H8BA	0.2149	0.2696	0.5090	0.092*	
H8BB	0.1889	0.3706	0.4843	0.092*	
H8BC	0.2662	0.3478	0.5724	0.092*	
C9B	0.2642 (8)	0.3146 (8)	0.3089 (9)	0.075 (3)	
H9BA	0.2160	0.2699	0.3197	0.112*	
H9BB	0.3093	0.2925	0.2564	0.112*	
H9BC	0.2364	0.3693	0.2815	0.112*	

### Atomic displacement parameters (Å^2^)

**Table:**

	*U*^11^	*U*^22^	*U*^33^	*U*^12^	*U*^13^	*U*^23^
Pd1	0.0294 (2)	0.0285 (2)	0.0404 (3)	0.00255 (18)	−0.0012 (2)	0.00392 (19)
Pd2	0.0316 (3)	0.0303 (3)	0.0412 (3)	−0.00305 (18)	−0.0025 (2)	−0.0019 (2)
Se1A	0.0338 (3)	0.0395 (4)	0.0412 (4)	0.0011 (3)	−0.0012 (3)	−0.0027 (3)
Se1B	0.0422 (4)	0.0342 (4)	0.0424 (4)	−0.0013 (3)	0.0037 (3)	−0.0015 (3)
Cl1A	0.0511 (11)	0.0351 (9)	0.104 (2)	−0.0069 (8)	0.0006 (12)	0.0004 (10)
Cl1B	0.0410 (10)	0.0524 (12)	0.111 (2)	0.0080 (9)	0.0036 (12)	0.0005 (13)
N1A	0.034 (3)	0.037 (3)	0.056 (4)	0.013 (2)	0.000 (3)	0.003 (3)
N1B	0.043 (3)	0.040 (3)	0.052 (4)	−0.015 (3)	−0.003 (3)	−0.003 (3)
C1A	0.033 (3)	0.037 (4)	0.042 (4)	−0.001 (3)	0.010 (3)	−0.002 (3)
C2A	0.056 (4)	0.046 (4)	0.043 (4)	0.001 (3)	0.008 (4)	0.007 (3)
C3A	0.069 (6)	0.051 (5)	0.047 (5)	−0.007 (4)	0.006 (4)	0.004 (4)
C4A	0.070 (6)	0.063 (6)	0.052 (5)	−0.013 (5)	0.017 (5)	−0.006 (4)
C5A	0.047 (4)	0.052 (5)	0.075 (6)	0.000 (4)	0.017 (4)	−0.002 (4)
C6A	0.034 (4)	0.046 (4)	0.055 (5)	0.002 (3)	0.000 (3)	−0.003 (3)
C7A	0.028 (3)	0.051 (4)	0.053 (4)	0.010 (3)	−0.003 (3)	0.001 (4)
C8A	0.065 (6)	0.063 (6)	0.064 (6)	0.030 (5)	−0.005 (5)	0.012 (5)
C9A	0.057 (6)	0.051 (5)	0.071 (7)	0.020 (4)	0.009 (5)	−0.008 (4)
C1B	0.043 (4)	0.031 (3)	0.046 (4)	0.002 (3)	0.004 (3)	0.006 (3)
C2B	0.042 (4)	0.055 (4)	0.047 (5)	0.000 (3)	−0.006 (3)	0.006 (4)
C3B	0.055 (5)	0.070 (6)	0.049 (5)	0.011 (5)	−0.008 (4)	0.005 (4)
C4B	0.073 (7)	0.057 (5)	0.065 (6)	0.012 (5)	0.011 (5)	0.019 (5)
C5B	0.058 (5)	0.049 (4)	0.059 (5)	0.006 (4)	0.003 (4)	0.017 (4)
C6B	0.051 (5)	0.030 (3)	0.058 (5)	−0.001 (3)	0.009 (4)	−0.001 (3)
C7B	0.061 (5)	0.029 (3)	0.057 (5)	−0.009 (3)	0.006 (4)	−0.003 (3)
C8B	0.055 (5)	0.065 (6)	0.063 (6)	−0.022 (5)	0.011 (5)	0.001 (5)
C9B	0.077 (7)	0.086 (8)	0.062 (6)	−0.051 (6)	−0.008 (5)	−0.013 (6)

### Geometric parameters (Å, °)

**Table:**

Pd1—N1A	2.186 (6)	C5A—H5AA	0.9300
Pd1—Cl1A	2.335 (2)	C6A—C7A	1.505 (12)
Pd1—Se1A	2.3858 (9)	C7A—H7AA	0.9700
Pd1—Se1A^i^	2.4043 (8)	C7A—H7AB	0.9700
Pd1—Se1B^ii^	3.4307 (9)	C8A—H8AA	0.9600
Pd2—N1B	2.180 (6)	C8A—H8AB	0.9600
Pd2—Cl1B	2.341 (2)	C8A—H8AC	0.9600
Pd2—Se1B	2.3872 (9)	C9A—H9AA	0.9600
Pd2—Se1B^i^	2.4021 (8)	C9A—H9AB	0.9600
Pd2—Se1A^iii^	3.4317 (9)	C9A—H9AC	0.9600
Se1A—C1A	1.905 (8)	C1B—C2B	1.391 (12)
Se1A—Pd1^i^	2.4043 (8)	C1B—C6B	1.393 (12)
Se1B—C1B	1.928 (8)	C2B—C3B	1.402 (13)
Se1B—Pd2^i^	2.4021 (8)	C2B—H2BA	0.9300
N1A—C7A	1.468 (11)	C3B—C4B	1.345 (15)
N1A—C9A	1.474 (12)	C3B—H3BA	0.9300
N1A—C8A	1.486 (12)	C4B—C5B	1.400 (15)
N1B—C8B	1.477 (12)	C4B—H4BA	0.9300
N1B—C7B	1.487 (12)	C5B—C6B	1.406 (12)
N1B—C9B	1.488 (12)	C5B—H5BA	0.9300
C1A—C2A	1.393 (11)	C6B—C7B	1.493 (13)
C1A—C6A	1.425 (11)	C7B—H7BA	0.9700
C2A—C3A	1.419 (12)	C7B—H7BB	0.9700
C2A—H2AA	0.9300	C8B—H8BA	0.9600
C3A—C4A	1.388 (15)	C8B—H8BB	0.9600
C3A—H3AA	0.9300	C8B—H8BC	0.9600
C4A—C5A	1.367 (15)	C9B—H9BA	0.9600
C4A—H4AA	0.9300	C9B—H9BB	0.9600
C5A—C6A	1.404 (13)	C9B—H9BC	0.9600
			
N1A—Pd1—Cl1A	95.12 (19)	C1A—C6A—C7A	119.0 (7)
N1A—Pd1—Se1A	91.53 (18)	N1A—C7A—C6A	112.2 (6)
Cl1A—Pd1—Se1A	173.08 (7)	N1A—C7A—H7AA	109.2
N1A—Pd1—Se1A^i^	172.88 (18)	C6A—C7A—H7AA	109.2
Cl1A—Pd1—Se1A^i^	91.99 (6)	N1A—C7A—H7AB	109.2
Se1A—Pd1—Se1A^i^	81.38 (3)	C6A—C7A—H7AB	109.2
N1A—Pd1—Se1B^ii^	97.4 (2)	H7AA—C7A—H7AB	107.9
Cl1A—Pd1—Se1B^ii^	99.90 (8)	N1A—C8A—H8AA	109.5
Se1A—Pd1—Se1B^ii^	81.06 (3)	N1A—C8A—H8AB	109.5
Se1A^i^—Pd1—Se1B^ii^	80.97 (3)	H8AA—C8A—H8AB	109.5
N1B—Pd2—Cl1B	95.2 (2)	N1A—C8A—H8AC	109.5
N1B—Pd2—Se1B	91.72 (19)	H8AA—C8A—H8AC	109.5
Cl1B—Pd2—Se1B	172.69 (7)	H8AB—C8A—H8AC	109.5
N1B—Pd2—Se1B^i^	172.78 (19)	N1A—C9A—H9AA	109.5
Cl1B—Pd2—Se1B^i^	91.97 (7)	N1A—C9A—H9AB	109.5
Se1B—Pd2—Se1B^i^	81.09 (3)	H9AA—C9A—H9AB	109.5
N1B—Pd2—Se1A^iii^	97.4 (2)	N1A—C9A—H9AC	109.5
Cl1B—Pd2—Se1A^iii^	100.11 (9)	H9AA—C9A—H9AC	109.5
Se1B—Pd2—Se1A^iii^	81.17 (3)	H9AB—C9A—H9AC	109.5
Se1B^i^—Pd2—Se1A^iii^	80.83 (3)	C2B—C1B—C6B	122.1 (8)
C1A—Se1A—Pd1	88.4 (2)	C2B—C1B—Se1B	121.8 (6)
C1A—Se1A—Pd1^i^	107.9 (2)	C6B—C1B—Se1B	116.0 (6)
Pd1—Se1A—Pd1^i^	97.39 (3)	C1B—C2B—C3B	118.7 (8)
C1B—Se1B—Pd2	87.8 (2)	C1B—C2B—H2BA	120.7
C1B—Se1B—Pd2^i^	108.2 (2)	C3B—C2B—H2BA	120.7
Pd2—Se1B—Pd2^i^	97.66 (3)	C4B—C3B—C2B	120.4 (9)
C7A—N1A—C9A	108.7 (7)	C4B—C3B—H3BA	119.8
C7A—N1A—C8A	109.4 (7)	C2B—C3B—H3BA	119.8
C9A—N1A—C8A	107.7 (7)	C3B—C4B—C5B	121.2 (9)
C7A—N1A—Pd1	112.5 (4)	C3B—C4B—H4BA	119.4
C9A—N1A—Pd1	111.3 (5)	C5B—C4B—H4BA	119.4
C8A—N1A—Pd1	107.1 (5)	C4B—C5B—C6B	120.2 (9)
C8B—N1B—C7B	107.6 (7)	C4B—C5B—H5BA	119.9
C8B—N1B—C9B	107.2 (8)	C6B—C5B—H5BA	119.9
C7B—N1B—C9B	109.0 (8)	C1B—C6B—C5B	117.3 (8)
C8B—N1B—Pd2	112.8 (6)	C1B—C6B—C7B	121.2 (8)
C7B—N1B—Pd2	112.9 (5)	C5B—C6B—C7B	121.5 (8)
C9B—N1B—Pd2	107.1 (6)	N1B—C7B—C6B	111.9 (7)
C2A—C1A—C6A	120.6 (7)	N1B—C7B—H7BA	109.2
C2A—C1A—Se1A	122.9 (6)	C6B—C7B—H7BA	109.2
C6A—C1A—Se1A	116.5 (6)	N1B—C7B—H7BB	109.2
C1A—C2A—C3A	119.5 (8)	C6B—C7B—H7BB	109.2
C1A—C2A—H2AA	120.2	H7BA—C7B—H7BB	107.9
C3A—C2A—H2AA	120.2	N1B—C8B—H8BA	109.5
C4A—C3A—C2A	119.1 (9)	N1B—C8B—H8BB	109.5
C4A—C3A—H3AA	120.5	H8BA—C8B—H8BB	109.5
C2A—C3A—H3AA	120.5	N1B—C8B—H8BC	109.5
C5A—C4A—C3A	121.6 (9)	H8BA—C8B—H8BC	109.5
C5A—C4A—H4AA	119.2	H8BB—C8B—H8BC	109.5
C3A—C4A—H4AA	119.2	N1B—C9B—H9BA	109.5
C4A—C5A—C6A	121.0 (9)	N1B—C9B—H9BB	109.5
C4A—C5A—H5AA	119.5	H9BA—C9B—H9BB	109.5
C6A—C5A—H5AA	119.5	N1B—C9B—H9BC	109.5
C5A—C6A—C1A	118.1 (8)	H9BA—C9B—H9BC	109.5
C5A—C6A—C7A	123.0 (8)	H9BB—C9B—H9BC	109.5
			
N1A—Pd1—Se1A—C1A	60.9 (3)	Pd1—Se1A—C1A—C2A	113.8 (7)
Cl1A—Pd1—Se1A—C1A	−103.4 (7)	Pd1^i^—Se1A—C1A—C2A	16.6 (7)
Se1A^i^—Pd1—Se1A—C1A	−119.7 (2)	Pd1—Se1A—C1A—C6A	−65.4 (6)
Se1B^ii^—Pd1—Se1A—C1A	158.1 (2)	Pd1^i^—Se1A—C1A—C6A	−162.6 (5)
N1A—Pd1—Se1A—Pd1^i^	168.8 (2)	C6A—C1A—C2A—C3A	−0.7 (13)
Cl1A—Pd1—Se1A—Pd1^i^	4.5 (7)	Se1A—C1A—C2A—C3A	−179.9 (7)
Se1A^i^—Pd1—Se1A—Pd1^i^	−11.87 (5)	C1A—C2A—C3A—C4A	−1.7 (14)
Se1B^ii^—Pd1—Se1A—Pd1^i^	−94.00 (3)	C2A—C3A—C4A—C5A	1.3 (15)
N1B—Pd2—Se1B—C1B	−60.7 (3)	C3A—C4A—C5A—C6A	1.5 (16)
Cl1B—Pd2—Se1B—C1B	101.4 (7)	C4A—C5A—C6A—C1A	−3.9 (14)
Se1B^i^—Pd2—Se1B—C1B	120.0 (2)	C4A—C5A—C6A—C7A	176.2 (9)
Se1A^iii^—Pd2—Se1B—C1B	−157.9 (2)	C2A—C1A—C6A—C5A	3.4 (12)
N1B—Pd2—Se1B—Pd2^i^	−168.8 (2)	Se1A—C1A—C6A—C5A	−177.4 (7)
Cl1B—Pd2—Se1B—Pd2^i^	−6.7 (7)	C2A—C1A—C6A—C7A	−176.6 (7)
Se1B^i^—Pd2—Se1B—Pd2^i^	11.94 (5)	Se1A—C1A—C6A—C7A	2.6 (10)
Se1A^iii^—Pd2—Se1B—Pd2^i^	93.95 (3)	C9A—N1A—C7A—C6A	70.6 (9)
Cl1A—Pd1—N1A—C7A	163.2 (5)	C8A—N1A—C7A—C6A	−172.0 (7)
Se1A—Pd1—N1A—C7A	−14.9 (6)	Pd1—N1A—C7A—C6A	−53.1 (8)
Se1A^i^—Pd1—N1A—C7A	−20 (2)	C5A—C6A—C7A—N1A	−105.4 (9)
Se1B^ii^—Pd1—N1A—C7A	−96.1 (5)	C1A—C6A—C7A—N1A	74.7 (10)
Cl1A—Pd1—N1A—C9A	41.0 (6)	Pd2—Se1B—C1B—C2B	−112.6 (7)
Se1A—Pd1—N1A—C9A	−137.1 (6)	Pd2^i^—Se1B—C1B—C2B	−15.2 (7)
Se1A^i^—Pd1—N1A—C9A	−142.1 (15)	Pd2—Se1B—C1B—C6B	66.1 (6)
Se1B^ii^—Pd1—N1A—C9A	141.7 (6)	Pd2^i^—Se1B—C1B—C6B	163.5 (6)
Cl1A—Pd1—N1A—C8A	−76.5 (6)	C6B—C1B—C2B—C3B	1.8 (13)
Se1A—Pd1—N1A—C8A	105.4 (6)	Se1B—C1B—C2B—C3B	−179.5 (6)
Se1A^i^—Pd1—N1A—C8A	100.4 (16)	C1B—C2B—C3B—C4B	0.2 (14)
Se1B^ii^—Pd1—N1A—C8A	24.2 (6)	C2B—C3B—C4B—C5B	−0.3 (15)
Cl1B—Pd2—N1B—C8B	−39.8 (7)	C3B—C4B—C5B—C6B	−1.6 (15)
Se1B—Pd2—N1B—C8B	137.9 (6)	C2B—C1B—C6B—C5B	−3.6 (12)
Se1B^i^—Pd2—N1B—C8B	144.0 (14)	Se1B—C1B—C6B—C5B	177.7 (6)
Se1A^iii^—Pd2—N1B—C8B	−140.7 (6)	C2B—C1B—C6B—C7B	175.2 (8)
Cl1B—Pd2—N1B—C7B	−162.1 (5)	Se1B—C1B—C6B—C7B	−3.5 (11)
Se1B—Pd2—N1B—C7B	15.6 (6)	C4B—C5B—C6B—C1B	3.4 (13)
Se1B^i^—Pd2—N1B—C7B	22 (2)	C4B—C5B—C6B—C7B	−175.4 (9)
Se1A^iii^—Pd2—N1B—C7B	97.0 (6)	C8B—N1B—C7B—C6B	−74.1 (9)
Cl1B—Pd2—N1B—C9B	77.9 (7)	C9B—N1B—C7B—C6B	170.0 (7)
Se1B—Pd2—N1B—C9B	−104.4 (7)	Pd2—N1B—C7B—C6B	51.1 (8)
Se1B^i^—Pd2—N1B—C9B	−98.3 (17)	C1B—C6B—C7B—N1B	−72.6 (10)
Se1A^iii^—Pd2—N1B—C9B	−23.1 (7)	C5B—C6B—C7B—N1B	106.2 (9)

Symmetry codes: (i) −*x*+1, −*y*+1, *z*; (ii) *x*, *y*, *z*+1; (iii) −*x*+1, −*y*+1, *z*−1.

### Hydrogen-bond geometry (Å, °)

**Table:**

*D*—H···*A*	*D*—H	H···*A*	*D*···*A*	*D*—H···*A*
C5A—H5AA···Cl1A^iv^	0.93	2.91	3.782 (10)	156.
C7A—H7AA···Cl1A^iv^	0.97	2.94	3.853 (8)	158.
C9A—H9AC···Cl1A	0.96	2.79	3.325 (10)	116.
C5B—H5BA···Cl1B^v^	0.93	2.94	3.808 (11)	156.
C8B—H8BB···Cl1B	0.96	2.80	3.347 (11)	117.

Symmetry codes: (iv) *x*+1/2, −*y*+1/2, −*z*+2; (v) −*x*+1/2, *y*−1/2, −*z*+1.

## References

[bb1] Brown, M. J. & Corrigan, J. F. (2004). *J. Organomet. Chem.* **689**, 2872–2879.

[bb2] Chakraborty, T., Srivastava, K., Singh, H. B. & Butcher, R. J. (2011). *J. Organomet. Chem.* **696**, 2782–2788.

[bb3] Dey, S., Jain, V. K., Varghese, B., Schurr, T., Niemeyer, M., Kaim, W. & Butcher, R. J. (2006). *Inorg. Chim. Acta*, **359**, 1449–1457.

[bb4] Flack, H. D. (1983). *Acta Cryst.* A**39**, 876–881.

[bb5] Ford, S., Morley, C. P. & Di Viara, M. (2004). *Inorg. Chem.* **43**, 7101–7110. 10.1021/ic040069y 15500348

[bb6] Kaur, R., Menon, S. C., Panda, S., Singh, H. B., Patel, R. P. & Butcher, R. J. (2009). *Organometallics*, **28**, 2363–2371.

[bb7] Morley, C. P., Webster, C. A. & Di Vaira, M. (2006). *J. Organomet. Chem.* **691**, 4244–4249.

[bb8] Nakata, N., Uchiumi, R., Yoshino, T., Ikeda, T., Kamon, H. & Ishii, A. (2009). *Organometallics*, **28**, 1981–1984.

[bb9] Oilunkaniemi, R., Laitinen, R. S. & Ahlgrén, M. (1999). *J. Organomet. Chem.* **587**, 200–206.

[bb10] Oilunkaniemi, R., Laitinen, R. S. & Ahlgrén, M. (2000). *J. Organomet. Chem.* **595**, 232–240.

[bb11] Oilunkaniemi, R., Laitinen, R. S. & Ahlgrén, M. (2001). *J. Organomet. Chem.* **623**, 168–175.

[bb12] Oxford Diffraction (2007). *CrysAlis PRO* Oxford Diffraction Ltd, Abingdon, England.

[bb13] Sheldrick, G. M. (2008). *Acta Cryst.* A**64**, 112–122. 10.1107/S0108767307043930 18156677

[bb14] Spek, A. L. (2009). *Acta Cryst.* D**65**, 148–155. 10.1107/S090744490804362X PMC2631630 19171970

